# Dual-Specificity Phosphatase 12 Targets p38 MAP Kinase to Regulate Macrophage Response to Intracellular Bacterial Infection

**DOI:** 10.3389/fimmu.2017.01259

**Published:** 2017-10-09

**Authors:** Sharol Su Lei Cho, Jian Han, Sharmy J. James, Chin Wen Png, Madhushanee Weerasooriya, Sylvie Alonso, Yongliang Zhang

**Affiliations:** ^1^Department of Microbiology and Immunology, Yong Loo Lin School of Medicine, Singapore, Singapore; ^2^Immunology Programme, Life Science Institute, National University of Singapore, Singapore, Singapore

**Keywords:** dual-specificity phosphatase, MAP kinases, inflammatory cytokine, macrophages, toll-like receptor signaling, bacterial infections

## Abstract

The mitogen-activated protein kinase (MAPK) cascades are activated in innate immune cells such as macrophages upon the detection of microbial infection, critically regulating the expression of proinflammatory cytokines and chemokines such as TNF-α, IL-6, and MCP-1. As a result, activation of MAPKs is tightly regulated to ensure appropriate and adequate immune responses. Dual-specificity phosphatases (DUSPs) are a family of proteins which specifically dephosphorylates threonine and tyrosine residues essential for MAPK activation to negatively regulate their activation. DUSP12 is a member of atypical DUSPs that lack MAPK-binding domain. Its substrate and function in immune cells are unknown. In this study, we demonstrated that DUSP12 is able to interact with all the three groups of MAPKs, including extracellular signal-regulated protein kinase, JNK, and p38. To investigate the function of DUSP12 in macrophages in response to TLR activation and microbial infection, we established RAW264.7 cell lines stably overexpressing DUSP12 and found that overexpression of DUSP12 inhibited proinflammatory cytokine and chemokine production in response to TLR4 activation, heat-inactivated *Mycobacterium tuberculosis* stimulation as well as infections by intracellular bacteria including *Listeria moncytogenesis* and *Mycobacterium bovis* BCG by specifically inhibiting p38 and JNK. In addition, a scaffold protein known as signal transducing adaptor protein 2 (STAP2), was found to mediate the interaction between DUSP12 and p38. Thus, DUSP12 is a bona fide MAPK phosphatase, playing an important role in MAPK-regulated responses to bacterial infection. Our study provides a model where atypical DUSPs regulate MAPKs *via* scaffold, thereby regulating immune responses to microbial infection.

## Introduction

The innate immune system is the first line of defense against microbe infection. It responses rapidly upon pathogen recognition and triggering inflammation. The pathogen-associated molecular patterns (PAMPs) derived from invading pathogens can be recognized by a family of transmembrane pattern recognition receptors (PRRs) called Toll-like receptors (TLRs) located on the cellular surface or endosomes of immune cells. There are 10 and 12 functional TLRs identified in human and mouse, respectively, each recognizing different ligands (1, 2). Pathogens may present various ligands which can be recognized by multiple TLRs. For instance, *Listeria monocytogenes*, a gram-positive intracellular bacterium, expresses a myriad of TLR ligands, including peptidoglycan, flagellin, and bacterial DNA. Therefore, recognition of peptidoglycan by a heterodimer of TLR2 and TLR6, lipoteichoic acid by TLR4, flagellin by TLR5, and bacterial DNA by TLR9 lead to the detection of invading *L. monocytogenes* by innate immune cells and activate the overall immune response (3–5).

Toll-like receptor activation leads to the initiation of two important signaling cascades, the MyD88-dependent and the MyD88-independent, which in turn, trigger proinflammatory responses (6). Both signaling cascades lead to the activation of the mitogen-activated protein kinases (MAPKs) which then activate transcription factor AP-1 (7–9). The MAPK cascade comprises of MAPK kinase kinases (MKKKs), MAPK kinases (MKKs), and MAPKs where their activation relies on sequential phosphorylation of these proteins (10). The activation of the three major MAPKs, namely the extracellular signal-regulated protein kinases (ERK), the p38 MAP kinases (p38), and the c-Jun NH2-terminal kinases (JNK), requires the phosphorylation of both threonine and tyrosine residues within the Thr-X-Tyr (TXY) motif in their activation loop. The intensity and duration of MAPK activation control the transcription, translation and mRNA stabilization of numerous proinflammatory cytokine and chemokine genes (11). Therefore, the magnitude and duration of MAPKs activation need to be tightly regulated to ensure controlled immune responses.

Dual-specificity phosphatases (DUSPs) are a family of protein phosphatases which can dephosphorylate both threonine and tyrosine residues in the Thr-X-Tyr (TXY) activation motif of MAPKs. Dephosphorylation negatively regulates the intensity and duration of MAPK activation (12, 13). DUSPs can be classified into classical or atypical DUSPs based on the presence of a MAPK binding domain (14). Classical DUSPs, also known as MAPK phosphatases (MKPs), contain a highly conserved C-terminal phosphatase domain and an N-terminal Cdc25 homology domain. The phosphatase domain shows similarity to the prototypic dual-specificity protein phosphatase VH-1 containing a signature catalytic motif “HCxxGxxR” (15). The Cdc25 homology domain is alternatively known as MAPK binding domain (MKB) and it consists of a kinase-interacting motif (KIM) which is crucial for docking to MAPKs (16). The atypical DUSPs, on the other hand, lack the N-terminal Cdc25 homology domain and have relatively simpler structure compared to classical DUSPs. Although phylogenetic analysis suggested diverse gene origins among atypical DUSPs, they all contain a conserved phosphatase catalytic site, identical to the one in classical DUSPs (17). Studies have shown that some atypical DUSPs can regulate the activation of MAPKs despite the lack of MAPK substrate recognition and binding domain (18, 19). However, functions of these atypical DUSPs in immune responses are largely unknown.

Dual-specificity phosphatase 12 is an atypical DUSP which is highly conserved among mammalian species (20). It contains a dual-specificity phosphatase catalytic domain, a C2H2 zinc finger domain, and an aldehyde dehydrogenase cysteine (ALDH) active site (21). Previous studies have shown that DUSP12 can dephosphorylate glucokinase, regulate cell cycle, and is involved in stress-induced cell death and cancer (22–24). In addition, it has been shown that DUSP12 is generally expressed in many immune cells and tissues (25). However, its function in immune response is unknown. In this study, we demonstrated that DUSP12 interacts with all three groups of MAPKs in HEK293T cells despite the lack of MKB and the interaction between DUSP12 and p38 was mediated by signal transducing adaptor protein 2 (STAP2). Further, we established DUSP12 stably overexpressing RAW264.7 cell lines and studied the function of this protein in macrophages. Overexpression of DUSP12 suppressed the production of various proinflammatory cytokines and chemokines upon TLR activation or intracellular bacterial infection by *L. monocytogenes* or *Mycobacterium bovis* BCG by inactivating JNK and p38, and defective killing of intracellular pathogens.

## Materials and Methods

### Cell Culture

RAW264.7 cells and HEK293T cells were grown in complete Gibco^®^ RPMI Media 1640 and Gibco^®^ DMEM (Invitrogen), respectively, supplemented with 10% heat-inactivated fetal bovine serum (Invitrogen) and 1% penicillin/streptomycin (Invitrogen). The pcDNA control and DUSP12 stably overexpressed cell lines were grown in complete RPMI medium containing 500 mg/ml G418 (Sigma). All cells were grown in humidified incubator at 37°C with 5% CO_2_.

### Establishment of DUSP12 Stably Overexpressing RAW263.7 Cell Lines

The pcDNA3.1-DUSP12 and pcDNA3.1 empty vectors were transfected into RAW264.7 cells using Lipofectamine LTX reagents (Invitrogen) following the manufacturer’s protocol. Transfected cells were maintained in complete medium containing 500 mg/ml G418 and the G418 containing medium was replenished for every three days. After two weeks of selection, individual colony was isolated and subjected to screening *via* quantitative real-time PCR (qRT-PCR) and Western blot to obtain DUSP12 overexpressing cells. Primers used to clone DUSP12 into pcDNA vector: forward primer 5′-TGAAGGAAGTGGGCCAATAG-3′ and reverse primer 5′-GACCACAGGAGCACTGTTCA-3′.

### Molecular Biology

FLAG-tagged constructs were made using the plasmid pXJ40 backbone (26). The following primers were used to construct the FLAG-tagged proteins: forward primer 5′-CCGGATCCATGATGGCGAAAAAGC-3′ and reverse primer5′-GGGAATTCTCATGCATTGTGTGGATTTTTCTTAAAC-3′ for full-length STAP1; forwards primer 5′-GGGGATCCATGGCCACAGCCCTGA-3′ and reverse primer 5′-GGGAATTCTCAGTGTTCCAGTGCCCTCC-3′ for full-length STAP2.

### Dual Luciferase Reporter Assay

2 × 10^5^ RAW264.7 cells were cotransfected with 50 ng AP-1 reporter construct, 400 ng pcDNA-vector, -DUSP12, and -DUSP12-PM together with 10 ng of pRL-null plasmid using Lipofectamine LTX reagent (Invitrogen). Equal amounts of pcDNA3.1 empty vector were transfected as control. Reporter activity was determined with Promega Dual Luciferase Assay System (Promega). Firefly luciferase values were normalized for transfection efficiency by means of the *Renilla luciferase* activity that is constitutively expressed by pRL-null.

### Quantitative Real-time PCR

Total mRNA was extracted using Trizol (Invitrogen) and cDNA was synthesized using ImProm-II™ Reverse Transcription System (Promega). qRT-PCR was performed using CFX connect Real Time System. Relative expression levels of DUSP12, TNF-α, IL-6, IL-1β, MCP-1, and IL-10 were calculated by ΔΔCt method and normalized to β-actin. The following mouse specific primers were used: forward primer 5′-TGAAGGAACTGGGCCAATAG-3′and reverse primer 5′-GACCACAGGAGCACTGTTCA-3′ for DUSP12, forward primer 5′-TCCCAGGTTCTCTTCAAGGGA-3′ and reverse primer 5′-GGTGAGGAGCACGTAGTCGG-3′for TNF-α, forward primer 5′-GATGCTACCAAACTGGATATAATC-3′and reverse primer 5′-TGTACTCCAGGTAGCTATG-3′ for IL-6, forward primer 5′-CAACCAACAAGTGATATTCTCCATG-3′ and reverse primer 5′-ATCCACACTCTCCAGCTGCA-3′ for IL-1β, forward primer 5′-CTCAGCCAGATGCAGTTAACGCCC-3′reverse primer 5′-GGTGCTGAAGACCTTAGGGCAGAT-3′for MCP-1, forward primer 5′-GCTCTTACTGACTGGCATGAG-3′ and reverse primer 5′-CGCAGCTCTAGGAGCATGTG-3′ for IL-10, forward primer 5′-AGCACTGTGTTGGCATAGAGGTC-3′reverse primer 5′-CTTCTTGGGTATGGAATCCTGTG-3′for β-actin.

### Enzyme-Linked Immunosorbent Assay (ELISA)

The concentrations of IL-6 and TNF-α in culture supernatants were determined by IL-6 and TNF-α ELISA kits (BD Pharmingen™). MCP-1 concentration was determined using the MCP-1 ELISA kit (eBioscience).

### Western Blot

Proteins were harvested using protein lysis buffer (1 M Tris–Cl, 5 M NaCl, 100% NP-40) containing 1× Halt Protease and Phosphatase Inhibitor Cocktail and 0.5 M EDTA (Thermo scientific). Protein samples were subjected to SDS-PAGE and Western blot was performed using the following antibodies: anti-DUSP12 (Santa Cruz, 1:500 dilution), anti- beta actin (Cell signaling, 1:1,000 dilution), anti-ERK (Cell Signaling, 1:1,000 dilution), antiphosphorylated-ERK(Sigma, 1:1,000 dilution), anti-JNK(Cell Signaling, 1:1,000 dilution), antiphosphorylated-JNK (Sigma, 1:1,000 dilution), anti-p38 (Cell Signaling, 1:1,000 dilution), or antiphosphorlated-p38 (Sigma, 1:1,000 dilution). Primary antibodies were detected with either antigoat (Santa Cruz, 1:4,000 dilution) or anti-rabbit IgG (Cell Signaling, 1:4,000 dilution) horseradish peroxidase-coupled secondary antibodies. Images were captured using Amersham^®^ Hyperfilm^®^ ECL™ and MP Autoradiography Films (GE Healthcare).

### Immunoprecipitation

Flag-tagged or HA-tagged vectors were transiently transfected or cotransfected into 293T cells using Mirus. Proteins were harvested using protein lysis buffer (1 M Tris–Cl, 5 M NaCl, 100% NP-40) containing 1× Halt Protease and Phosphatase Inhibitor Cocktail and 0.5 M EDTA (Thermo scientific) 36 h after transfection. From the protein lysate, 12.5% was used for WCL, while 75% was used for immunoprecipitation. Protein lysates were incubated with anti-Flag M2 beads overnight at 4°C on a shaker. After the protein binding M2 beads had been washed extensively using lysis buffer, 4× loading dye was added to resuspend the M2 beads and elute bound proteins. The samples were subjected to SDS-PAGE and Western blot was performed with respective antibodies.

### *Listeria* Intracellular Killing Assay

The cells were washed twice with PBS and infected with *L. monocytogenes* at the bacteria-to-cell ratio 10:1 in antibiotics-free RPMI medium. After 1 h infection, cells were washed with PBS twice to remove extracellular *L. monocytogenes*. Cells were incubated in RPMI medium containing 5 μg/ml gentamicin for another 6 or 12 h and lysed by 200 μl 0.2% Triton X-100 in distilled water. The lysates containing *L. monocytogenes* were plated on Brain Heart Infusion (BHI) agar plates. The BHI agar plates were incubated overnight at 37°C and colony forming units (CFU) were counted.

### *Mycobacterium* Intracellular Infection Assay

The macrophages were seeded a day prior to infection at a cell density of 5 × 10^4^ cells/well. Infection was conducted at a multiplicity-of-infection 8 using *M. bovis BCG*. After an hour of infection, cells were washed with PBS to remove extracellular bacteria and media was replaced with RPMI media containing 10% fetal bovine serum. BCG was harvested at various time-points—days 0, 1, 2, 5, and 7. Cells were lysed with 0.1% Triton X-100 and lysates were plated on Middlebrook’s 7H11 agar plates. The plates were incubated at 37°C for 16 days and colony forming units (CFU) were recorded.

### Heat-Inactivated *Mycobacterium tuberculosis* Stimulation Assay

The macrophages were seeded a day prior to infection at a cell density of 1.5 × 10^6^ cells/well. Infection was conducted at a multiplicity-of-infection 8 using heat-inactivated *M. tuberculosis*. Cells were treated with Trizol at 3 and 6 h post-stimulation to conduct qRT-PCR to assess cytokine expression. Cell supernatant was collected to measure cytokine expression *via* ELISA.

### Cell Proliferation Assay

The cells were washed twice with PBS and infected with *L. monocytogenes* at the bacteria-to-cell ratio 10:1 in antibiotics-free RPMI medium. After 1 h infection, cells were washed twice to remove extracellular Listeria. Cells were incubated in RPMI medium containing 5 μg/ml gentamicin for another 24 or 48 h. At respective time-points, cells were washed twice with PBS to remove dead floating cells. Cell proliferation was determined by crystal violet staining. Absorbance was measured at 590 nm. Percentage of cell survival was obtained by comparing absorbance of infected to that of uninfected cells.

### Statistical Analysis

Data were expressed as the mean ± SE. Data comparisons were made with Student’s *t*-test or two-way ANOVA using Microsoft Excel and GraphPad PRISM. Differences were considered significant when the *P* value is <0.05. *P*-values <0.05, 0.01, and 0.001 were represented by “*,” “**,” and “***,” respectively.

## Results

### DUSP12 Interacted with MAPKs and Inhibited AP-1 Promoter Activity

**Figure 1:**
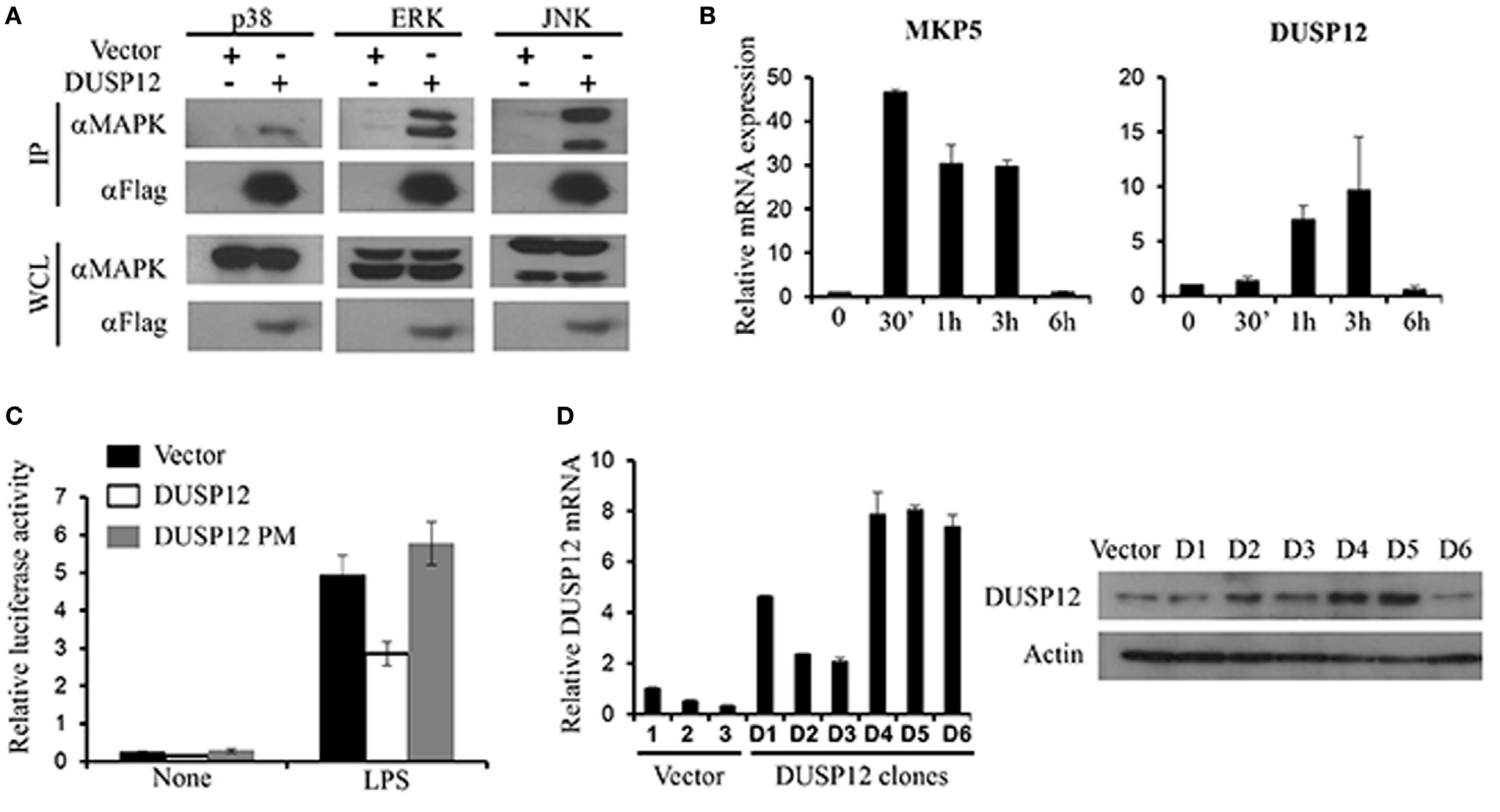
Dual-specificity phosphatase 12 (DUSP12) interacted with mitogen-activated protein kinases (MAPKs) *in vitro* and inhibit AP-1 activation. **(A)** HEK293T cells were transfected with the pXJ40-Flag-DUSP12 or empty vectors. Flag-DUSP12 was pulled down using anti-Flag M2 resin. Flag-DUSP12 in the immunoprecipitates (IP) and in the whole cell lysates (WCL) were detected by anti-Flag Western blot analysis. Copurified extracellular signal-regulated protein kinase (ERK), Jun NH2-terminal kinase (JNK), and p38 in IP and total p38, JNK, and ERK in the WCL were detected using anti-P38, anti-JNK, and anti-ERK Western blot analysis. **(B)** RAW264.7 cells were stimulated with 100 ng/ml LPS. MKP5 and DUSP12 mRNA expression was determined by quantitative real-time PCR (qRT-PCR). **(C)** Overexpression of DUSP12 in RAW264.7 cells inhibited AP-1 promoter activity. Point mutation in the phosphatase catalytic site abolished the inhibition. **(D)** DUSP12 mRNA and protein expression in DUSP12 stably transfected RAW264.7 cells. The data shown are representative of three independent experiments with similar results.

Mitogen-activated protein kinase-binding domain is essential for MAPK interaction and substrate specificity in typical DUSPs. Despite the absence of the binding domain, atypical DUSPs, such as DUSP14, had demonstrated marked ability in dephosphorylating MAPKs (18). Similarly, DUSP12 lacks the MKB domain and thus we examined DUSP12’s ability to interact with MAPKs. Mouse full-length DUSP12 was cloned into pcDNA vectors and transfected into HEK293T cells to examine its ability to bind endogenously to MAPKs. We found that DUSP12 was able to interact with endogenous p38, ERK, and JNK (Figure 1A). To examine the role of DUSP12 in innate immunity, we first examined the expression of DUSP12 in RAW264.7 cells, an immortalized murine macrophage cell line, in response to LPS stimulation. The activation of TLR4 will initiate a downstream MAPK signaling cascade which eventually activates transcription factor, AP-1. MKP5, a typical DUSP, serves as a positive control where its expression and function in macrophages have been examined in an earlier study (27). We found that LPS stimulation resulted in the upregulation of DUSP12 expression in macrophages (Figure 1B), although the upregulation appeared to be weaker as compared to that of MKP5. To examine if DUSP12 regulates MAPK-mediated response in immune cells, we performed a dual-luciferase assay by cotransfecting pcDNA vector carrying murine DUSP12 cDNA sequence and a vector consisting of the promoter construct of transcription factor AP-1 conjoined to the luciferase gene. The DUSP12 construct with a point mutation in the catalytic site serves as a negative control. As shown in Figure 1C, overexpression of functional DUSP12 inhibited AP-1 promoter activity in response to LPS stimulation, whereas the DUSP12 mutant lost the inhibition (Figure 1C). On the other hand, DUSP12 overexpression does not affect NFκB promoter activity in response to LPS stimulation (Figure S1 in Supplementary Material). These evidences indicate that DUSP12 could have utilized its phosphatase domain to regulate MAPK signaling and MAPK-mediated responses. To further investigate the function of DUSP12 in macrophages, we generated stable overexpression of DUSP12 in RAW264.7 macrophage cell line. The overexpression of DUSP12 in selected clones were confirmed at both mRNA and protein levels (Figure 1D). Clone number 5 (D5) with the highest DUSP12 expression was selected for subsequent experiments to elucidate the role of this molecule in innate immunity.

### DUSP12 Inhibits Proinflammatory Mediator Expression and p38/JNK Activation upon TLR4 Activation

**Figure 2:**
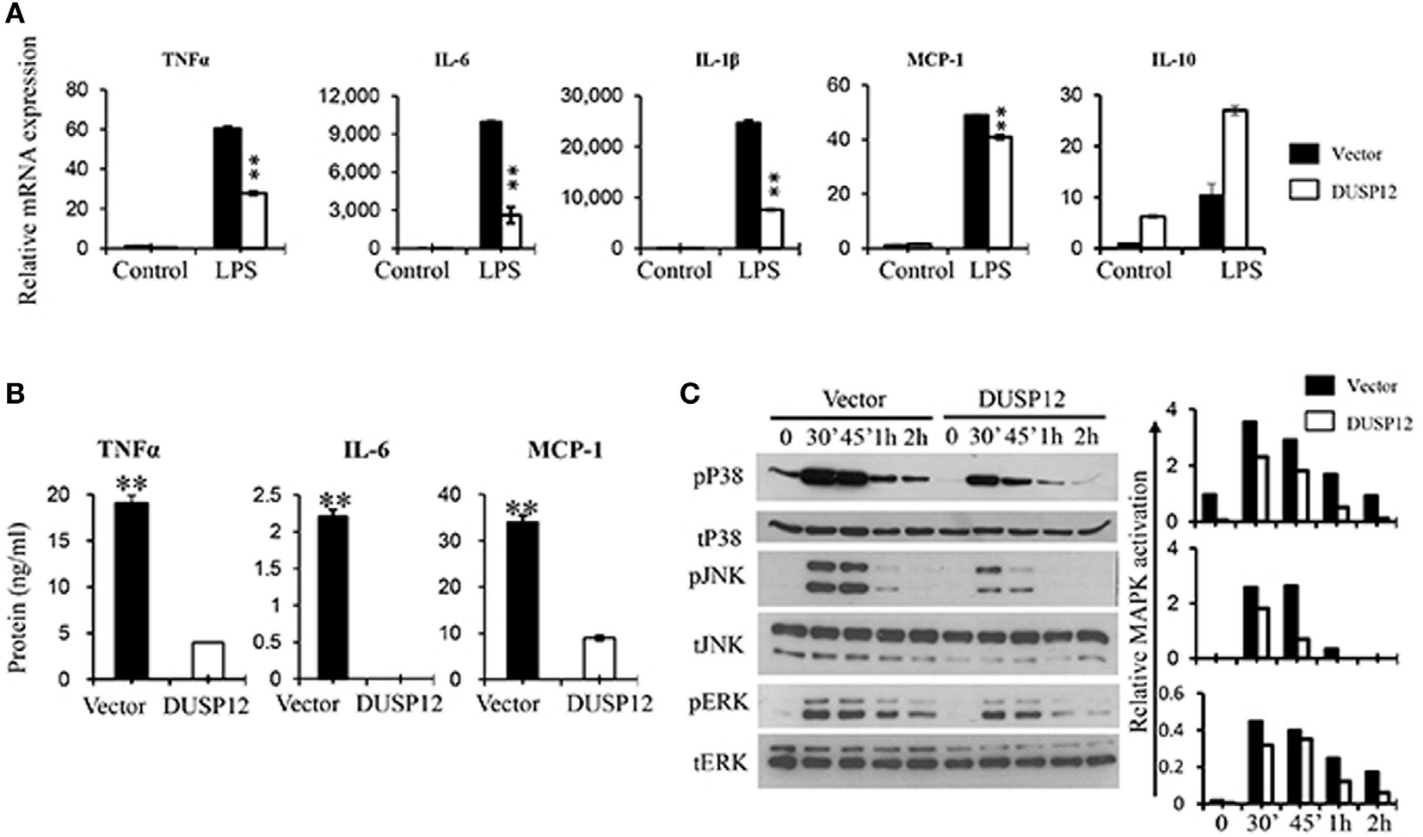
Overexpression of dual-specificity phosphatase 12 (DUSP12) inhibited proinflammatory cytokine and chemokine production in response to toll-like receptor 4 (TLR4) activation by dephosphorylating p38 and Jun NH2-terminal kinase (JNK) mitogen-activated protein kinases (MAPKs). DUSP12 overexpressing and pcDNA control RAW264.7 cells were stimulated with 100 ng/ml LPS. **(A)** Total mRNA was isolated after 3 h LPS stimulation and subjected to quantitative real-time PCR (qRT-PCR) analysis of TNF-α, IL-6, IL-1β, MCP-1, and IL-10 level. **(B)** Culture supernatants were harvested 24 h after LPS stimulation for enzyme-linked immunosorbent assay (ELISA) analysis of TNF-α, IL-6, and MCP-1 level. **(C)** Total proteins were harvested at different time-points for the detection of p38, JNK, and extracellular signal-regulated protein kinase (ERK) activation by Western blot. Relative p38, JNK, and ERK activation was measured by comparing phospho-MAPKs to total-MAPKs. The data shown are representative of two to three independent experiments with similar results. Data are presented as mean ± SEM. *P-value < 0.05; **P-value < 0.01.

Macrophages are known to secrete proinflammatory mediators including cytokines and chemokines upon activation of their pattern recognition receptors in the event of pathogen infection. To examine whether DUSP12 regulates the expression of these proinflammatory mediators in TLR4 signaling, we stimulated DUSP12 overexpressing (D5) and vector control RAW264.7 cells with LPS and found that mRNA expression of TNF-α, IL-6, IL-1β, and MCP-1 were highly induced in both D5 and control cells by LPS (Figure 2A). Moreover, there was a significant reduction in the expression of these proinflammatory mediators in D5 compared to the control cells. Interestingly, the expression of IL-10, a potent anti-inflammatory cytokine, was enhanced in D5 cells (Figure 2A). Similar phenotype was observed at protein levels for TNF-α, IL-6 and MCP-1, where the proteins detected were suppressed in D5 when compared to the control (Figure 2B). Together, these results indicate the inhibitory role of DUSP12 on the expression of proinflammatory mediators in macrophages in response to TLR activation.

Mitogen-activated protein kinase pathway plays a crucial role in regulating the production of cytokines and chemokines during TLR activation in macrophages. To examine if the inhibition of proinflammatory cytokine production by DUSP12 is due to its regulation on MAPKs, the MAPKs activation profile in D5 and control cells were determined. In response to LPS stimulation, p38, JNK, and ERK were rapidly phosphorylated in both D5 and control cells but to different extents (Figure 2C). Overexpression of DUSP12 demonstrated significant reduction in p38 and JNK activation, while slight reduction in the activation of ERK was observed (Figure 2C). Together, the results demonstrated that DUSP12 inhibited proinflammatory cytokines and chemokines production in macrophages in response to LPS, possibly through inactivation of p38 and JNK.

To validate the function of DUSP12 in regulation of MAPK activation and cytokine expression in response to LPS, we utilized siRNA approach to knockdown the expression of DUSP12 in macrophages. We achieved about 50% knockdown of DUSP12 protein expression using a pool of siRNA targeting 4 regions of *Dusp12* gene (Figure S2A in Supplementary Material). Increased activation of p38, JNK, and ERK in response to LPS stimulation was observed in cells with DUSP12 knockdown (Figure S2B in Supplementary Material), which is associated with increased expression of proinflammatory cytokines including TNF-α and MCP-1 (Figure S2C in Supplementary Material). These results confirmed the negative regulation of MAPK activation and proinflammatory cytokine expression in macrophages by DUSP12.

### DUSP12 Inhibits Proinflammatory Mediator Expression and p38 Activation upon Heat-Inactivated *Mycobacterium* Stimulation

Macrophages serve as a first line of defense during infection and thus, these pathogens evolved a myriad of strategies to evade macrophage-mediated inflammatory responses. It is vital to understand the regulation of these responses to better control bacterial infections. As such, DUSP12 function in regulating inflammatory responses is studied; where intracellular pathogens were used to stimulate macrophages to assess the proinflammatory cytokine production and MAPK activation profile. D5 and control cells were activated by heat-inactivated *M. tuberculosis* to examine macrophage inflammatory responses. Consistent with the role of DUSP12 in TLR4-mediated cytokine expression, overexpression of DUSP12 suppressed the expression of TNF-α, IL-1β, and MCP-1 (Figure S3A in Supplementary Material). This is also reflected in protein expression of TNF-α and MCP-1, where cytokine levels were significantly reduced in D5 cells as compared to vector control cells (Figure S3B in Supplementary Material).

**Figure 3:**
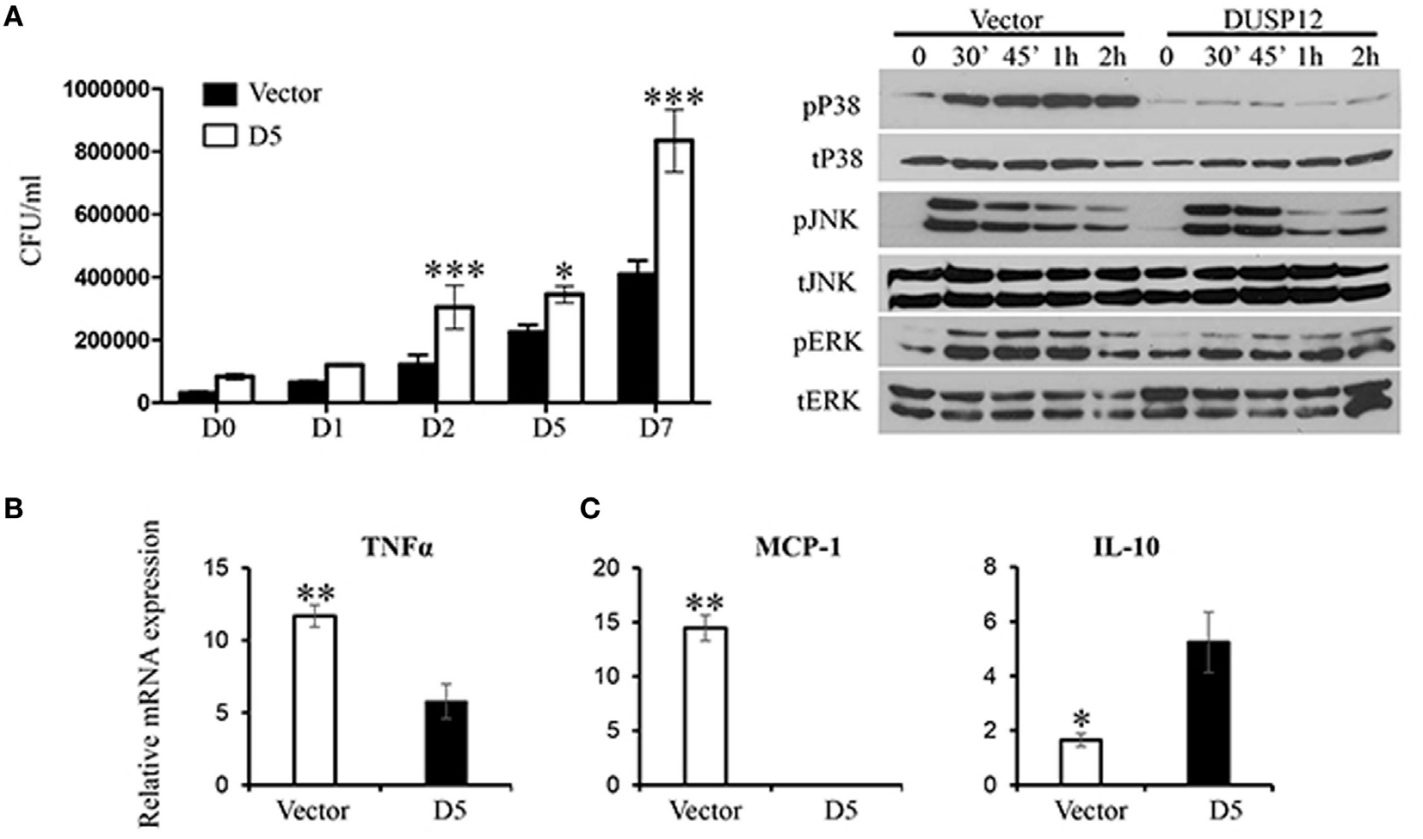
Overexpression of dual-specificity phosphatase 12 (DUSP12) inhibited proinflammatory cytokine and chemokine production in response to *Mycobacteria* infection by dephosphorylating p38 and Jun NH2-terminal kinase (JNK) mitogen-activated protein kinases (MAPKs). DUSP12 overexpressing and pcDNA control RAW264.7 cells were infected by *Mycobacterium BCG* at the bacteria-to-cell ratio 8:1 for 1 h and extracellular *Mycobacterium bovis* BCG was removed. Infected macrophages were incubated in RPMI medium, containing 10% FBS for indicated periods of time. **(A)** Macrophages were lysed at various time-points using 0.1% Triton-X—days 0, 1, 2, 5, and 7 to release intracellular *M. bovis* BCG and plated on Middlebrook’s 7H11 agar medium. Colony forming unit (CFU) counts were enumerated after 16-day incubation and statistical analysis was conducted. Results are represented as the means ± SDs of triplicates. Results are representative of two independent experiments. *P-value < 0.05 and ***P-value < 0.001. **(B)** Total proteins were harvested at different time-points for the detection of p38, JNK, and extracellular signal-regulated protein kinase (ERK) activation by Western blot. Relative p38, JNK, and ERK activation was measured by comparing phospho-MAPKs to total-MAPKs. **(C)** Total mRNA was isolated 6 h post-*M. bovis* BCG infection and subjected to quantitative real-time PCR (qRT-PCR) analysis of TNF-α, MCP-1, and IL-10 levels. The data shown are representative of three to four independent experiments with similar results.

Thereafter, *M. bovis* BCG was used as a surrogate for *M. tuberculosis* in the macrophage stimulation set-ups. D5 and control macrophages were infected at a multiplicity of eight and bacteria growth was assessed by the comparing the number of colony forming units (CFU) at various time-points postinfection. The CFU counts demonstrated that DUSP12 overexpression resulted in higher bacteria burden across all time-points, where the difference is significant in the later time-points as compared to the control cells (Figure 3A). The phenotype observed was correlated with MAPK activation profile and cytokine production. Western blot analysis demonstrated that overexpression of DUSP12 greatly inhibited p38 activation, but not the activation of JNK or ERK upon BCG infection (Figure 3B). The cytokine profile in D5 and control cells upon BCG stimulation revealed that expression of proinflammatory cytokines including TNF-α and MCP-1 was suppressed while anti-inflammatory cytokine, IL10 was upregulated (Figure 3C). Together, these results suggest that DUSP12 negatively regulates p38 activation and proinflammatory cytokine expression in macrophages upon the detection of Mycobacteria.

### DUSP12 Inhibits Proinflammatory Mediator Expression and p38 Activation in Response to *Listeria* Infection

**Figure 4:**
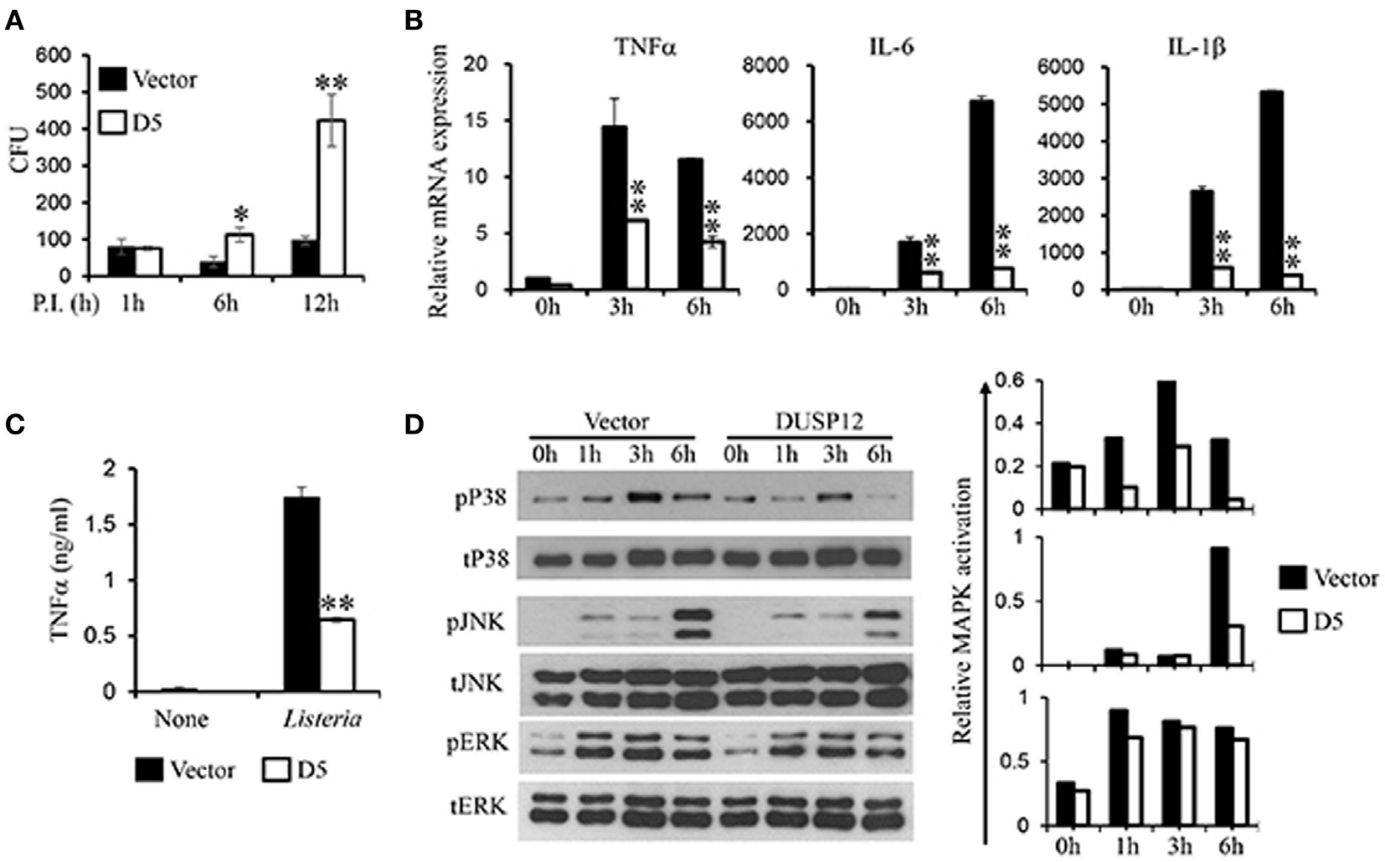
Overexpression of dual-specificity phosphatase 12 (DUSP12) impaired intracellular killing of *Listeria monocytogenes* in RAW264.7 cells. DUSP12 overexpressing and pcDNA control RAW264.7 cells were infected with *L. monocytogenes* at bacteria-to-cell ratio of 10:1 for 1 h and extracellular Listeria were removed. Infected cells were continued to be incubated in RPMI medium with 5 μg/ml gentamicin for indicated time periods. **(A)** Six and 12-h postinfection, cells were lysed to release intracellular *L. monocytogenes*. The released *L. monocytogenes* were subsequently cultured on BMI agar plates to count colony forming unit (CFU) after overnight incubation. **(B)** Total mRNA was isolated 3 and 6 h post-*Listeria* infection and subjected to quantitative real-time PCR (qRT-PCR) analysis of TNF-α, IL-6, and IL-1β levels. **(C)** Culture supernatants were harvested 24 h after *Listeria* stimulation for enzyme-linked immunosorbent assay (ELISA) analysis of TNF-α. **(D)** Total proteins were harvested at different time-points for the detection of p38, JNK, and extracellular signal-regulated protein kinase (ERK) activation by Western blot. Relative p38, JNK, and ERK activation was measured by comparing phospho-MAPKs to total-MAPKs. The data shown are representative of three to four independent experiments with similar results. Data are presented as mean ± SEM. *P-value < 0.05; **P-value < 0.01.

In the same manner, macrophages play a crucial role in immune responses against *L. monocytogenes*. To examine the regulatory role of DUSP12 in macrophages in response to *Listeria* infection, D5 and control cells were infected with *L. monocytogenes* at a MOI of 10 to assess bacterial load and cell survival. Similar to BCG infection profile, *Listeria* CFU counts was significantly higher in D5 cells as compared to the control at 6 and 12 h postinfection (Figure 4A). The higher bacteria load had also resulted in greater cell death, where the difference between D5 and control is pronounced at the 48 h postinfection (Figure S4 in Supplementary Material). Furthermore, mRNA and protein expression of proinflammatory cytokines including TNF-α, IL-6, IL-1β, and IFNβ were assessed. In control macrophages, the expression of these cytokines was greatly induced, while D5 cells showed significant suppression at 3 and 6 h postinfection (Figure 4B). In addition, TNF-α protein expression was significantly attenuated in DUSP12 overexpressing cells compared to that in control cells upon *Listeria* infection (Figure 4C).

Next, the activation of MAPKs in D5 and control cells in response to *Listeria* infection was examined. All three groups of MAPKs were activated in response to *Listeria* infection (Figure 4D). Clearly, overexpression of DUSP12 greatly inhibited p38 activation at all time points examined, compared to the control cells. In addition, JNK activation at 6 h postinfection was reduced by DUSP12 overexpression, whereas ERK activation was comparable across all time-points between D5 and control cells (Figure 4D). Together, these results suggest that DUSP12 inhibits inflammatory mediator expression in macrophages during Listeria infection through attenuation of p38, and to a lesser extent, JNK activation.

Next, we selected another overexpressing clone (OXE), which has DUSP12 levels of approximately three to four times that of the control cells (Figure S5A in Supplementary Material), to verify the function of DUSP12 in macrophages in response to intracellular bacterial infection. Similar to D5 (which has eightfolds of DUSP12 overexpression, Figure 1D), OXE cells has reduced inflammatory cytokine expression (Figure S5B in Supplementary Material), associated with reduced MAPK activation but to a lesser extend as D5 (Figure S5C in Supplementary Material), and were less able to control BCG replication compared to control cells (Figure S5D in Supplementary Material). Together, these results confirm the inhibitory function of this protein on macrophage responses to intracellular bacterial infection by targeting MAPKs.

### STAP2 Mediates the Interaction between DUSP12 and p38

**Figure 5:**
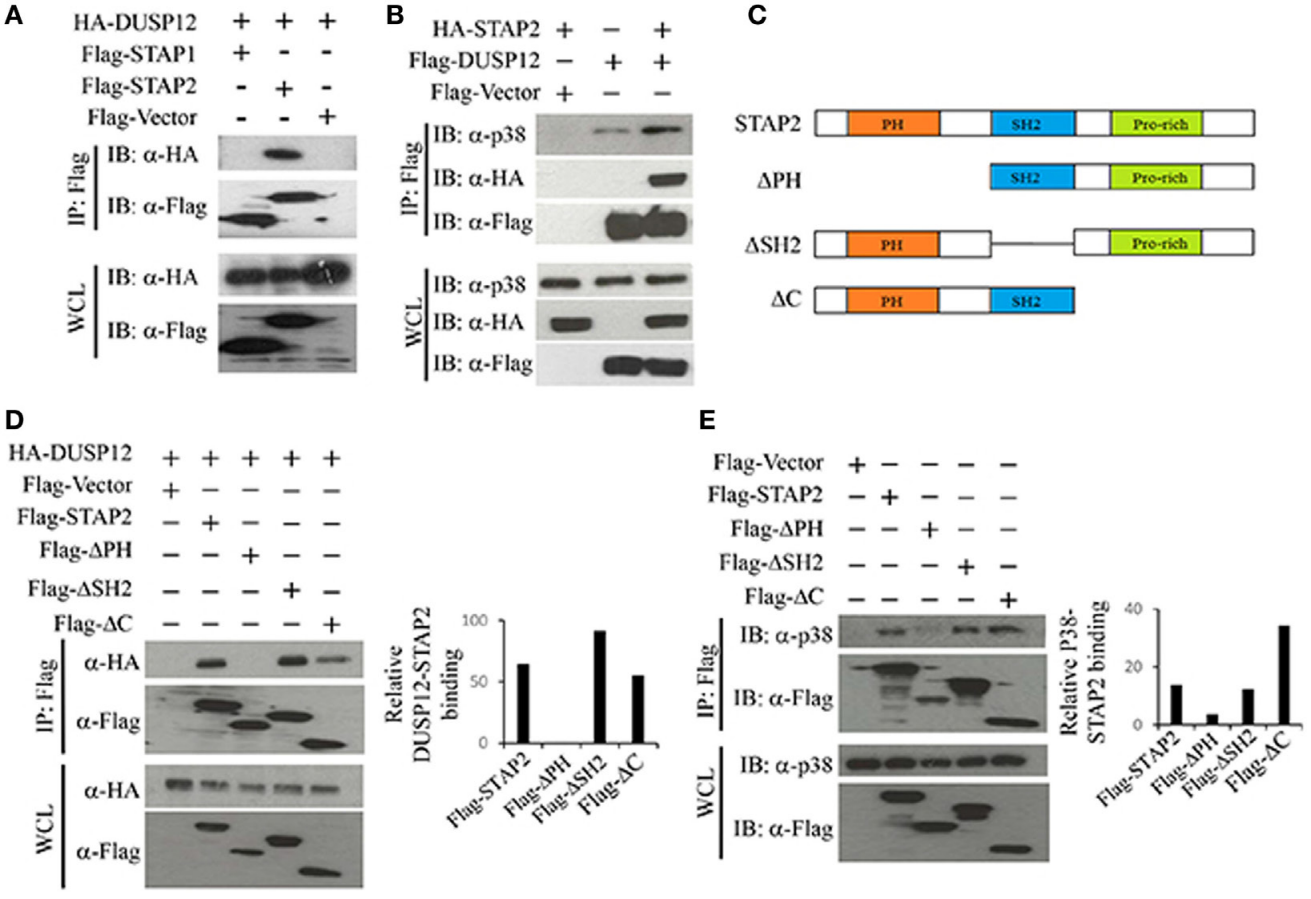
Signal transducing adaptor protein 2 (STAP2) enhanced interaction between dual-specificity phosphatase 12 (DUSP12) and p38 *in vitro*. **(A)** HEK293T cells were cotransfected with HA-DUSP12 and the pXJ40-Flag, pXJ40-Flag-STAP1, or pXJ40-Flag-STAP2 vectors. Flag-STAP1/2 was pulled down using anti-Flag M2 resin. Flag-STAP1 and Flag-STAP2 in the immunoprecipitates (IPs) and in the whole cell lysates (WCL) were detected by anti-Flag Western blot analysis. Copurified HA-DUSP12 in the IP and in the WCL was detected using anti-HA Western blot analysis. **(B)** HEK293T cells were cotransfected with HA-STAP2 and the pXJ40-Flag or pXJ40-Flag-DUSP12 vectors. Flag-DUSP12 was pulled down using anti-Flag M2 resin. Flag-DUSP12 in the IP and in the WCL were detected by anti-Flag Western blot analysis. Copurified p38 and HA-STAP2 in the IP and in the WCL were detected using anti-p38 and anti-HA Western blot analysis. **(C)** Schematic describing various STAP2 deletion constructs—full-length STAP2, ΔPH, ΔSH2, and ΔC. **(D)** HEK293T cells were cotransfected with HA-DUSP12 and the pXJ40-Flag, pXJ40-Flag-STAP2 or pXJ40-Flag-ΔPH, pXJ40-Flag-ΔSH2, and pXJ40-Flag-ΔC vectors. Flag-STAP constructs were pulled down using anti-Flag M2 resin. Flag-STAP constructs in the IP and in the WCL were detected by anti-Flag Western blot analysis. Copurified HA-DUSP12 in the IP and in the WCL was detected using anti-HA Western blot analysis. Relative DUSP12-STAP2 interaction is measured by quantification of HA-DUSP12 in the immunoprecipitate with respect to the levels of FLAG-STAP2 constructs in the immunoprecipitate. **(E)** HEK293T cells were cotransfected with the pXJ40-Flag, pXJ40-Flag-STAP2 or pXJ40-Flag-ΔPH, pXJ40-Flag-ΔSH2, and pXJ40-Flag-ΔC vectors. Flag-STAP2 constructs were pulled down using anti-Flag M2 resin. Flag-STAP2 constructs in the IP and in the WCL were detected by anti-Flag Western blot analysis. Copurified p38 were detected using anti-p38 Western blot analysis. Relative p38-STAP2 interaction is measured by quantification of p38 in the immunoprecipitate with respect to the levels of FLAG-STAP2 constructs in the immunoprecipitate. The data shown are representative of two to three independent experiments with similar results.

Dual-specificity phosphatase 12 was able to interact with endogenous p38, ERK, and JNK (Figure 1A), with stronger binding to ERK and JNK than to p38. DUSP12 is an atypical DUSP that lacks the MAPK binding domain. Therefore, we postulated that an additional binding agent is recruited for the interaction between DUSP12 and MAPKs. The binding of DUSP12 to p38 is the weakest among the three MAPKs (Figure 1A), yet DUSP12 has the strongest inhibition toward the activation of p38 compared with that of JNK or ERK in macrophages upon TLR activation or intracellular bacterial infection. Therefore, we speculated that scaffold proteins might be present to mediate the interaction between DUSP12 and p38. Studies by Sekine et al. (28) demonstrated that signal-transducing adaptor protein-2 (STAP-2) in macrophages modulates cytokine production in TLR4 signaling pathway and thus, we studied the possible role of STAP proteins (STAP1 and STAP2) in facilitating DUSP12 and p38 interaction. Coimmunoprecipitation of the STAP proteins with DUSP12 revealed that STAP2, and not STAP1, interacted with DUSP12 (Figure 5A). In addition, in the presence of STAP2, DUSP12, and p38 interaction was enhanced (Figure 5B). These results suggest that STAP2 may function as a scaffold protein which mediates the interaction between DUSP12 and p38.

Signal transducing adaptor protein 2 contains an N-terminal pleckstrin homology (PH) domain, a central Src homology 2-like (SH2) domain, and a proline-rich YXXQ motif in the C-terminal. Several studies demonstrated that the SH2 domain is important for binding to other proteins including FAK, IKKs, MyD88, and STAT3/5 (28–30). Hence, several STAP2 constructs were generated (Figure 5C) and the binding potential of these proteins to DUSP12 and p38 were examined. We found that deletion of the SH2 domain (ΔSH2) and C-terminal domain (ΔC) of STAP2 did not affect its interaction with DUSP12, whereas deletion of the PH domain (ΔPH) led to much weaker interaction with DUSP12 (Figure 5D). Similarly, ΔSH2 mutant and ΔC mutant of STAP2 were able to interact with p38, whereas ΔPH mutant failed to interact with p38 (Figure 5E). Together, these results suggest that the PH domain of STAP2 is required for its interaction with both DUSP12 and p38.

**Figure 6:**
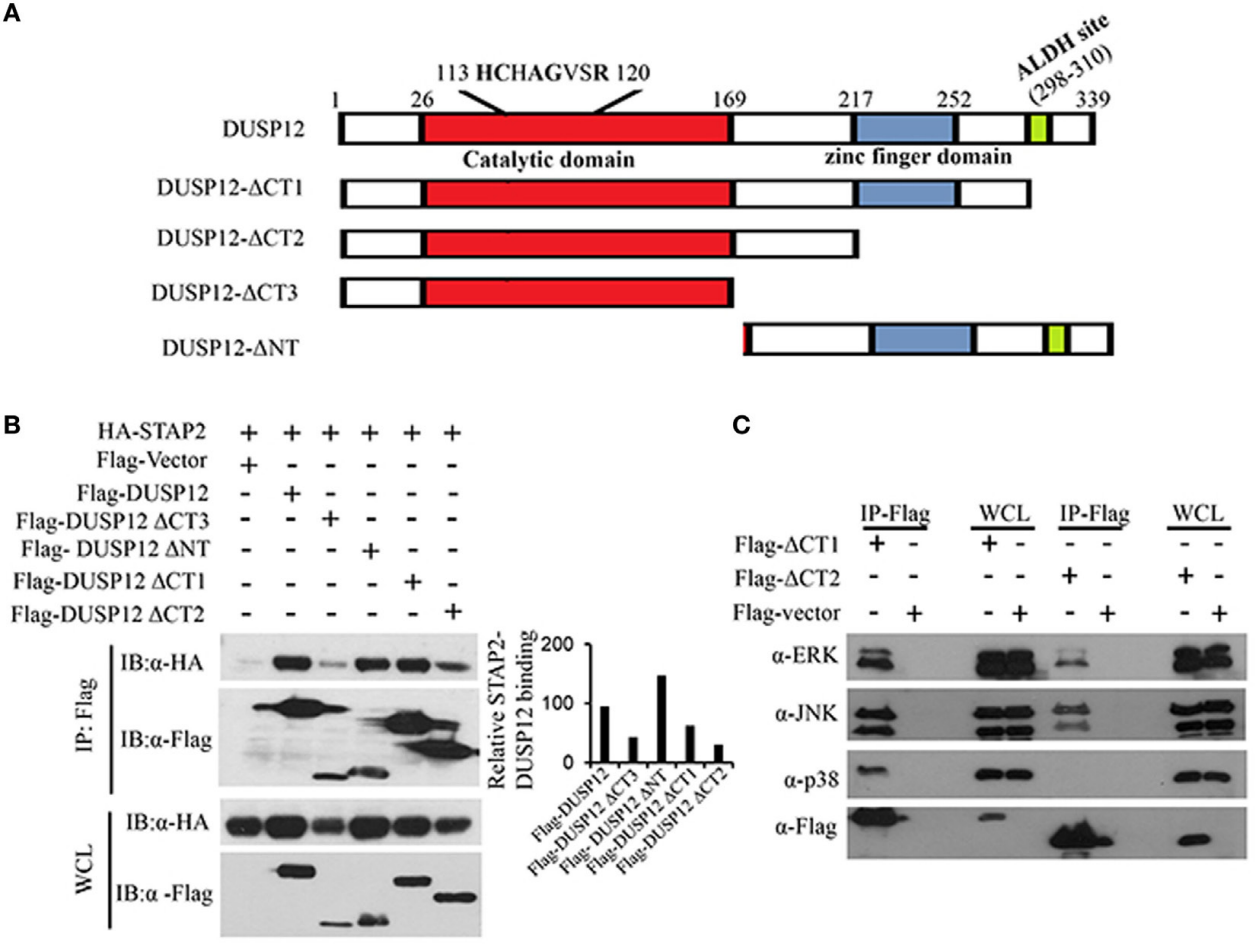
Pleckstrin homology (PH) domain of signal-transducing adaptor protein-2 (STAP2) is responsible for its interaction with p38 and dual-specificity phosphatase 12 (DUSP12) *in vitro*. **(A)** Schematic describing various DUSP12 deletion constructs. **(B)** HEK293T cells were transfected with HA-STAP2 and the pXJ40-Flag, pXJ40-Flag-DUSP12 or pXJ40-Flag-DUSP12-ΔCT1, pXJ40-Flag-DUSP12-ΔCT2, pXJ40-Flag-DUSP12-ΔCT3, or pXJ40-Flag-DUSP12-ΔNT vectors. Flag-tagged STAP2 mutants were pulled down using anti-Flag M2 resin. Flag-tagged STAP2 mutants in the immunoprecipitates (IP) and in the whole cell lysates (WCL) were detected by anti-Flag Western blot analysis. Relative STAP2-DUSP12 interaction is measured by quantification of HA-STAP2 in the immunoprecipitate with respect to the levels of FLAG-DUSP12 constructs in the immunoprecipitate. **(C)** HEK293T cells were cotransfected with pXJ40-Flag, pXJ40-Flag-DUSP12-ΔCT1, or pXJ40-Flag-DUSP12-ΔCT2 vectors. Flag-tagged DUSP12 mutants were pulled down using anti-Flag M2 resin. FLAG-STAP2 mutants in the IP and in the WCL were detected by anti-Flag Western blot analysis. Copurified extracellular signal-regulated protein kinase (ERK), p38, and JNK MAPKs in the IP and in the WCL were detected using anti-ERK, anti-JNK, and anti-p38 Western blot analysis. The data shown are representative of two to three independent experiments with similar results.

Dual-specificity phosphatase 12 contains an N-terminal catalytic domain, a C-terminal zinc finger domain, and an ALDH site (Figure 6A). To test the function of these domains of DUSP12 in its interaction with STAP2, we generated several truncated DUSP12 constructs (Figure 6A) and were used to perform immunoprecipitation with STAP2. We found that deletion of the N-terminal catalytic domain did not affect it interaction with STAP2, whereas deletion of its C-terminal domain, including the zinc finger domain and the ALDH site resulted in weakened interaction with STAP2 (Figure 6B). This suggests that the DUSP12 C-terminal domain including the zinc finger domain and the ALDH site is required for its interaction with STAP2. Furthermore, the lack of C-terminal domain in DUSP12 impaired its interaction with ERK, JNK, and p38 (Figure 6C). Particularly, the interaction between DUSP12 and p38 was completely lost, suggesting that the C-terminal domain of DUSP12 is required for its interaction with p38, likely *via* STAP2.

## Discussion

Mitogen-activated protein kinases control major cellular activities such as cell differentiation, stress responses, apoptosis, and immune defenses (31, 32) and thus it is of high importance to tightly regulate MAPK activity. DUSP plays a critical role in regulating these cascades, thereby keeping cellular activities in check. There are 25 DUSPs encoded by the human genome which comprises of 15 atypical DUSPs (14). Despite the abundance of atypical DUSPs, the biological functions of DUSPs are much less understood. In this study, we demonstrated that DUSP12, an atypical DUSP, acts as a p38 and JNK phosphatase in macrophages, thereby regulating macrophage responses to intracellular bacterial infection.

We first examined the expression of DUSP12 in macrophages in response to TLR activation and found that, unlike DUSP1 (MKP1) and DUSP10 (MKP5) which are rapidly induced in response to TLR activation (12, 27), the expression of DUSP12 was not as highly modulated in RAW264.7 cells (Figure 1B), suggesting that the expression of this gene is more tightly regulated than DUSP1 or DUSP10. Nevertheless, similar to DUSP10 (27), overexpression of DUSP12 inhibited the activation of AP-1, a transcription factor known be a downstream target of TLR4 signaling pathway and a major target of MAPKs, upon TLR4 activation (Figure 1C). Furthermore, the conserved phosphatase domain in DUSP12 is found to be crucial for its function in regulating AP-1 activation since point mutation in the enzymatic site abolished its inhibition of AP-1 activation. We further establish that DUSP12 can interact with the three major MAPKs groups—ERK, JNK, and p38, despite the lack of the MKB domain (Figure 1A). In addition, overexpression of DUSP12 in macrophages suppressed p38 and JNK activation in response to TLR4 stimulation or intracellular bacterial infection (Figures 2C, 3B and 4C). Together, these observations suggest that DUSP12 is a MAPK phosphatase and regulates MAPK-mediated responses, of which is dependent on its phosphatase activity.

Interestingly, immunoprecipitation data showed that the interaction between DUSP12 and p38 is the weakest as compared to JNK or ERK (Figure 1A), but it is apparent that DUSP12 mainly suppresses the activation of p38 in macrophages during intracellular bacterial infection (Figures 3B and 4D). This suggests that the presence of regulatory mechanism, which may mediate the interaction between DUSP12 and p38. It is well known that the formation of signaling complexes is crucial for the specificity of signal transduction cascades such as MAPK pathways. These complexes may be formed by direct docking interaction between corresponding partners in the signal cascade, or by assembly of signaling and regulatory components on scaffold proteins which bring their binding partners to specific substrates (10). DUSP can interact with MAPK either by docking or scaffolding. Classical DUSPs share a common structure comprising of a C-terminal catalytic domain and an N-terminal KBD, which contains conserved KIMs. They can recognize specific MAPK targets though interaction with a common docking (CD) site within their structure. Previous studies have shown that the KBD of DUSP10 binds p38α in a bipartite manner, in which two distinct helical regions of KBD engage the p38α docking site (33). However, atypical DUSP12, lacks the conserved KBD domain for docking with MAPKs. Therefore, alternative interactions may explain the specific inhibition of p38 and JNK activation. We propose the involvement of scaffold proteins which direct DUSP12 to its target MAPKs. To this end, we identified STAP2 as a scaffold protein mediating the interaction between DUSP12 and p38. We found that DUSP12 specifically interacts with STAP2 but not STAP1. In the presence of STAP2, p38 binding to DUSP12 is enhanced (Figures 5A,B), indicating STAP2 might recruit DUSP12 to p38. STAP2 is constitutively expressed in macrophages, and STAP2 deficient macrophages showed significantly impairment of TLR4-mediated cytokine production (28). Our results support previous literature, where STAP2 has a regulatory role in cellular signaling of innate and adaptive immune systems (34). We further identified that the PH domain of STAP2 is required for its interaction with DUSP12 and p38, whereas the C-terminal region of DUSP12 is important for its interaction with STAP2 and p38 (Figure 5). Despite these findings, the exact mechanism of STAP2–DUSP12 interaction with p38 is unclear. One possibility is that DUSP12 is constantly associated with STAP2, where the bound state restricts DUSP12 activity. Upon signaling from PRRs, DUSP12 may dissociate from STAP2 and form a complex with p38. Alternatively, cellular localization or specific extracellular stimuli may trigger the recruitment of DUSP12 to STAP2, which then form a complex with p38 to ensure controlled p38 activation.

Dual-specificity phosphatase 12 overexpressing RAW264.7 cells were generated to further explore the functions of the MAPK regulator. We found that the production of proinflammatory cytokines, TNF-α, IL-6 and MCP-1, were impaired in the DUSP12 overexpressing cells, associated with reduced activations of p38 and JNK, but not ERK activation in response to LPS stimulation, BCG, and *Listeria* infection (Figures 2–4).

Inflammatory activation of macrophages has been shown to be essential for defense against intracellular pathogens including *M. tuberculosis and L. monogeneses* (35). *M. tuberculosis* is the infectious agent of the fatal disease—tuberculosis. The bacilli enter the host *via* the respiratory tract and are met with alveolar macrophages. Current literature has reported various immune evasion techniques *M. tuberculosis* has employed and one of which is modulation of host cell signaling. Previous studies have described that activation of ERK and p38 pathways lead to the production of mycobacteria-induced TNF-α, while ERK activation alone results in mycobacteria induced MCP-1 (36, 37). As described in Figure 3B, the suppression of MAPK might have reduced the expression of the mycobacteria-induced TNF-α and MCP-1. However, contrary to the study by Song et al. (36), we observed that the p38 suppression by DUSP12 led to enhanced anti-inflammatory IL10 production in the mycobacteria stimulation model (Figure 3C). Our findings support that modulation of MAPK pathways leads to the suppression of antimycobacterial cytokines whilst enhancing the production of macrophage inhibitory cytokines (38). DUSP12 may facilitate *Mycobacteria* survival in host macrophages where we observed increase CFU counts in the DUSP12 overexpressing strain (Figure 3). With widespread incidences of multidrug resistant tuberculosis and limitations in novel tubercular drug discovery, DUSP12 may potentially serve as a target for drug development. As the mechanism to which DUSP12 regulates immune responses is unclear, we postulate that DUSP12 inhibition may favor inflammatory responses and thus enhancing bactericidal mechanisms. Akin to Mycobacteria, macrophages serve as a primary host for *L. monocytogenes*. Monocytes and macrophages play a key role in defense against *L. monocytogenes* infection (35), where TLR signaling is essential for protection. Monocyte- and macrophage-specific deficiency of TLR signaling resulted in susceptibility to *L. monocytogenes* infection in mice models, which are associated with defective production of proinflammatory cytokines, including IL-12 and TNF-α (35). These studies highlighted the importance of proper regulation of TLR signaling in innate immune cells such as macrophages in intracellular pathogen infection. In this study, we detected significant induction and secretion of TNF-α in macrophages in response to *Listeria* infection. Overexpression of DUSP12 impaired the expression of TNF-α by selectively inhibiting p38 and JNK (Figure 4). This demonstrates a negative role of DUSP12 in regulating macrophage responses against *Listeria* infection. Furthermore, we also demonstrated that overexpression of DUSP12 impaired the macrophage bactericidal mechanisms toward intracellular *Listeria*. Failure to control the infection leads to reduced cellular survival (Figure S2 in Supplementary Material). Together, our study suggests that DUSP12 plays an essential role in controlling intracellular bacteria replication through the regulation of MAPKs.

In summary, this study demonstrates that DUSP12 is a p38 and JNK phosphatase, where modulation in MAPKs activation leads to impairment of proinflammatory cytokine and chemokine production in macrophages upon TLR activation or pathogen infection. In addition, this study provides a model where atypical DUSPs, like DUSP12, regulate the activation of MAPKs *via* scaffold proteins to regulate immune responses despite the lack of a MAPK binding domain.

## Author Contributions

SC, HJ, SA and YZ designed the experiments and interpreted the data. CS, HJ and YZ wrote the manuscript. SC, HJ, SJ, CP and MW performed the study, data analysis and statistical evaluation.

## Conflict of Interest Statement

The authors declare that the research was conducted in the absence of any commercial or financial relationships that could be construed as a potential conflict of interest.

## Supplementary Material

Figure S1 DUSP12 does not inhibit NF_k_B activity. Overexpression of DUSP12 in RAW264.7 cells did not inhibit NF_k_B promoter activity. Point mutation in the phosphatase catalytic site showed a similar effect as the DUSP12 overexpressing and pcDNA control upon stimulation with 100 ng/ml LPS.

Figure S2 Knockdown of DUSP12 expression resulted in increased MAPK activation and increased expression of proinflammatory cytokines in response to LPS stimulation. **(A)** Total protein was harvested for the detection of DUSP12 protein expression in macrophages treated with 25 and 100 nM of NTC and 25, 50, and 100 nM of si-DUSP12. **(B)** RAW264.7 cells with control or si-DUSP12 treatment were stimulated with LPS. Total proteins were harvested at different time-points for the detection of p38, JNK and ERK activation by western-blot. Relative p38, JNK and ERK activation was measured by comparing phospho-MAPKs to actin. **(C)** Total mRNA was isolated 3-h post-LPS stimulation and subjected to quantitative qRT-PCR analysis of TNF-α and MCP-1. Results are represented as the means ± SDs of duplicates. Results are representative of two independent experiments. **P*-value < 0.05.

Figure S3 Overexpression of DUSP12 inhibited proinflammatory cytokine and chemokine production in response to *Mycobacteria* stimulation. DUSP12 overexpressing and pcDNA control RAW264.7 cells were stimulated with heat-inactivated *Mycobacterium tuberculosis* at the bacteria-to-cell ratio 8:1. **(A)** Total mRNA was isolated 3 and 6 h postinfection and subjected to quantitative qRT-PCR analysis of TNF-α, IL-1β, and MCP-1 levels. **(B)** Culture supernatants were harvested 24 h after heat-inactivated TB stimulation for ELISA analysis of TNF-α and MCP-1. Data are presented as mean ± SEM.

Figure S4 Cell survival after *L. monocytogenes* infection. DUSP12 overexpressing and pcDNA control RAW264.7 cells were infected with *L. monocytogenes* at bacteria-to-cell ratio of 10:1 for 1 h and extracellular Listeria were removed. Infected cells were continued to be incubated in RPMI medium with 5 μg/ml gentamicin for 24 or 48 h. Cell proliferation was determined by crystal violet staining. Absorbance was measured at 590 nm. Percentage of cell survival was obtained by comparing absorbance of infected to that of uninfected cells.

Figure S5 Threefold overexpression of DUSP12 demonstrates similar inhibition of proinflammatory cytokine and chemokine production in response to *M. bovis* BCG infection by dephosphorylating p38 and JNK MAPKs. **(A)** DUSP12 overexpressing clone, OXE demonstrates a 3-fold overexpression as compared to control vector. **(B)** OXE and pcDNA control RAW264.7 cells were infected by *Mycobacterium BCG* at the bacteria-to-cell ratio 8:1. Total proteins were harvested at different time-points for the detection of p38, JNK and ERK activation by western-blot. Relative p38, JNK and ERK activation was measured by comparing phospho-MAPKs to total-MAPKs. **(C)** Total mRNA was isolated 1 h post-*M. bovis* BCG infection and subjected to quantitative qRT-PCR analysis of TNF-α and MCP-1. **(D)** OXE and pcDNA control RAW264.7 cells were infected by *Mycobacterium BCG* at the bacteria-to-cell ratio 8:1 for 1 h and extracellular *M. bovis* BCG was removed. Infected macrophages were incubated in RPMI medium, containing 10% FBS for indicated periods of time. Macrophages were lysed at various time-points using 0.1% Triton-X—days 0, 1, 2, 5, and 7 to release intracellular *M. bovis* BCG and plated on Middlebrook’s 7H11 agar medium. CFU counts were enumerated after 16 days of incubation and statistical analysis was conducted. Results are represented as the means ± SDs of triplicates. Results are representative of two independent experiments. **P*-value < 0.05 and ****P*-value < 0.001.
