# Supplementary figures and images for: Dual-Specificity Phosphatase 12 Targets p38 MAP Kinase to Regulate Macrophage Response to Intracellular Bacterial Infection
Source: Front Immunol. 2017 Oct 9;8:1259. doi: 10.3389/fimmu.2017.01259 (PMC5640881; doi:10.3389/fimmu.2017.01259)

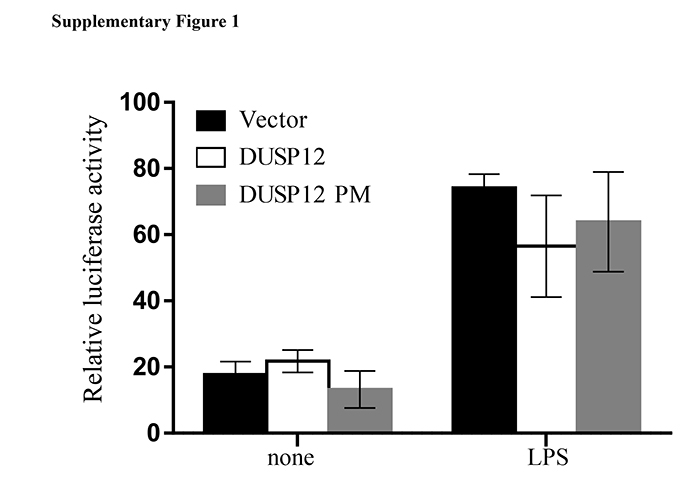

Supplement: Figure S1 — DUSP12 does not inhibit NFkB activity. Overexpression of DUSP12 in RAW264.7 cells did not inhibit NFkB promoter activity. Point mutation in the phosphatase catalytic site showed a similar effect as the DUSP12 overexpressing and pcDNA control upon stimulation with 100 ng/ml LPS. [file image_1.jpeg]

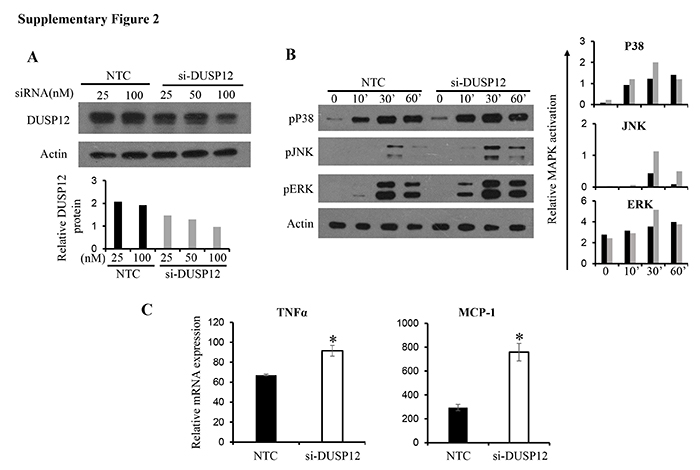

Supplement: Figure S2 — Knockdown of DUSP12 expression resulted in increased MAPK activation and increased expression of proinflammatory cytokines in response to LPS stimulation. (A) Total protein was harvested for the detection of DUSP12 protein expression in macrophages treated with 25 and 100 nM of NTC and 25, 50, and 100 nM of si-DUSP12. (B) RAW264.7 cells with control or si-DUSP12 treatment were stimulated with LPS. Total proteins were harvested at different time-points for the detection of p38, JNK and ERK activation by western-blot. Relative p38, JNK and ERK activation was measured by comparing phospho-MAPKs to actin. (C) Total mRNA was isolated 3-h post-LPS stimulation and subjected to quantitative qRT-PCR analysis of TNF-α and MCP-1. Results are represented as the means ± SDs of duplicates. Results are representative of two independent experiments. *P-value < 0.05. [file image_2.jpeg]

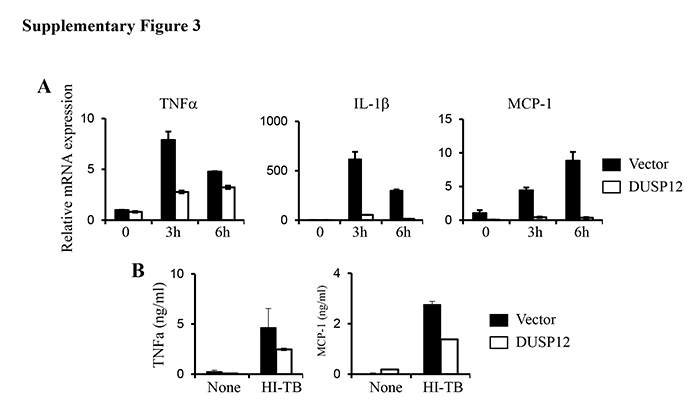

Supplement: Figure S3 — Overexpression of DUSP12 inhibited proinflammatory cytokine and chemokine production in response to Mycobacteria stimulation. DUSP12 overexpressing and pcDNA control RAW264.7 cells were stimulated with heat-inactivated Mycobacterium tuberculosis at the bacteria-to-cell ratio 8:1. (A) Total mRNA was isolated 3 and 6 h postinfection and subjected to quantitative qRT-PCR analysis of TNF-α, IL-1β, and MCP-1 levels. (B) Culture supernatants were harvested 24 h after heat-inactivated TB stimulation for ELISA analysis of TNF-α and MCP-1. Data are presented as mean ± SEM. [file image_3.jpeg]

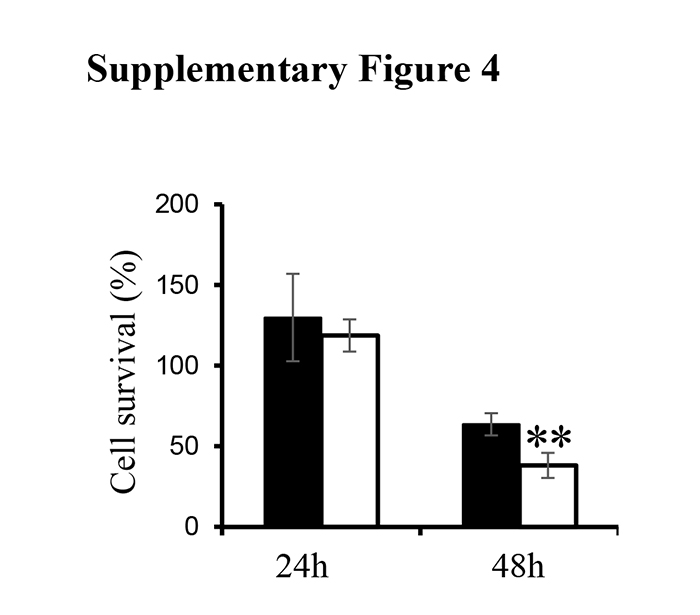

Supplement: Figure S4 — Cell survival after L. monocytogenes infection. DUSP12 overexpressing and pcDNA control RAW264.7 cells were infected with L. monocytogenes at bacteria-to-cell ratio of 10:1 for 1 h and extracellular Listeria were removed. Infected cells were continued to be incubated in RPMI medium with 5 μg/ml gentamicin for 24 or 48 h. Cell proliferation was determined by crystal violet staining. Absorbance was measured at 590 nm. Percentage of cell survival was obtained by comparing absorbance of infected to that of uninfected cells. [file image_4.jpeg]

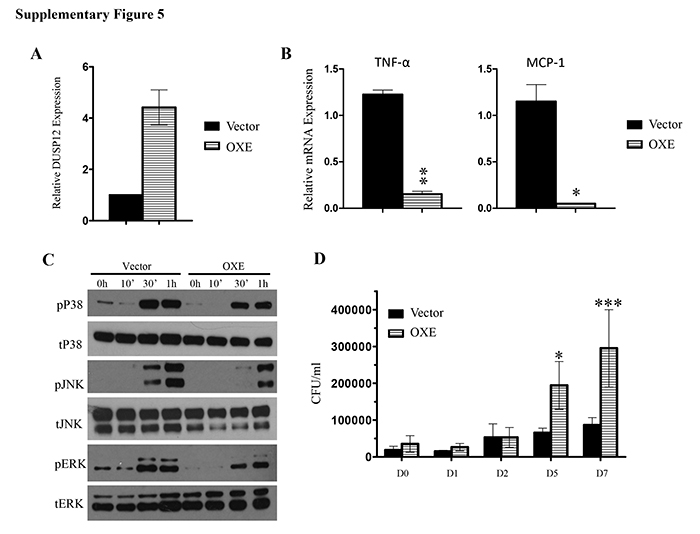

Supplement: Figure S5 — Threefold overexpression of DUSP12 demonstrates similar inhibition of proinflammatory cytokine and chemokine production in response to M. bovis BCG infection by dephosphorylating p38 and JNK MAPKs. (A) DUSP12 overexpressing clone, OXE demonstrates a 3-fold overexpression as compared to control vector. (B) OXE and pcDNA control RAW264.7 cells were infected by Mycobacterium BCG at the bacteria-to-cell ratio 8:1. Total proteins were harvested at different time-points for the detection of p38, JNK and ERK activation by western-blot. Relative p38, JNK and ERK activation was measured by comparing phospho-MAPKs to total-MAPKs. (C) Total mRNA was isolated 1 h post-M. bovis BCG infection and subjected to quantitative qRT-PCR analysis of TNF-α and MCP-1. (D) OXE and pcDNA control RAW264.7 cells were infected by Mycobacterium BCG at the bacteria-to-cell ratio 8:1 for 1 h and extracellular M. bovis BCG was removed. Infected macrophages were incubated in RPMI medium, containing 10% FBS for indicated periods of time. Macrophages were lysed at various time-points using 0.1% Triton-X—days 0, 1, 2, 5, and 7 to release intracellular M. bovis BCG and plated on Middlebrook’s 7H11 agar medium. CFU counts were enumerated after 16 days of incubation and statistical analysis was conducted. Results are represented as the means ± SDs of triplicates. Results are representative of two independent experiments. *P-value < 0.05 and ***P-value < 0.001. [file image_5.jpeg]
